# Crystal structure and Hirshfeld surface analysis of (μ-2-{4-[(carboxyl­atometh­yl)carbamo­yl]benz­amido}­acetato-κ^2^ *O*:*O*′)bis­[bis­(1,10-phenanthroline-κ^2^ *N*,*N*′)copper(II)] dinitrate *N*,*N*′-(1,4-phenyl­enedicarbon­yl)diglycine monosolvate octa­hydrate

**DOI:** 10.1107/S2056989019005164

**Published:** 2019-04-25

**Authors:** Niels-Patrick Pook, Arnold Adam, Mimoza Gjikaj

**Affiliations:** a Institute of Inorganic and Analytical Chemistry, Clausthal University of Technology, Paul-Ernst-Str. 4, D-38678, Clausthal-Zellerfeld, Germany

**Keywords:** crystal structure, copper(II) complex, *N*,*N*′-(1,4-phenyl­enedicarbon­yl)diglycine, phenanthroline ligand, supra­molecular inter­actions, Hirshfeld surface analysis.

## Abstract

The centrosymmetric binuclear complex cation of the title compound, [Cu_2_(C_12_H_10_N_2_O_6_)(C_12_H_8_N_2_)_4_](NO_3_)_2_·C_12_H_12_N_2_O_6_·8H_2_O, is composed of a Cu^II^ atom with a distorted trigonal–bipyramidal coordination environment defined by four N atoms from two bidentate 1,10-phenanthroline ligands and one oxygen atom from one-half of the monodentate *N*,*N*′-(1,4-phenyl­enedicarbon­yl)diglycinate anion. The asymmetric unit is completed by one-half of the *N*,*N*′-(1,4-phenyl­enedicarbon­yl)diglycine solvent mol­ecule, which is located on a centre of inversion, by one nitrate counter-anion and four water mol­ecules. In the crystal, the cationic complexes are linked *via* inter­molecular π–π stacking and through lone-pair⋯π inter­actions involving the *N*,*N*′-(1,4-phenyl­enedicarbon­yl)diglycinate anion and the phenanthroline ligands. The *N*,*N*′-(1,4-phenyl­enedicarbon­yl)diglycine solvent mol­ecule is involved in classical and non-classical hydrogen-bonding inter­actions, as well as π–π stacking inter­actions. The centroid-to-centroid distances between aromatic entities are in the range 3.5402 (5)–4.3673 (4) Å. The crystal structure is stabilized by further C—H⋯O contacts as well as by O—H⋯O and N—H⋯O hydrogen bonds between water mol­ecules, the nitrate anions, the *N*,*N*′-(1,4-phenyl­enedicarbon­yl)diglycinate ligands, *N*,*N*′-(1,4-phenyl­enedicarbon­yl)diglycine solvent mol­ecules and phenanthroline ligands, giving rise to a supra­molecular framework. A Hirshfeld surface analysis was carried out to qu­antify these inter­actions.

## Abstract 2

The Cu^II^ atom in the title compound has a distorted trigonal–bipyramidal coordination environment defined by four N atoms from two bidentate 1,10-phenanthroline ligands and one oxygen atom from one-half of the monodentate *N*,*N*′-(1,4-phenyl­enedicarbon­yl)diglycinate ligand. The dinuclear complex cations and the nitrate counter-anions as well as the solvate mol­ecules are linked by an intricate network of hydrogen bonds.

## Chemical context   

Over the past two decades, the syntheses and structural investigations of coordination polymers with different dimensions as well as metal–organic frameworks (MOFs) have attracted much attention because of their intriguing functional architectures and applications (Batten *et al.*, 2013 ▸; Leong & Vittal, 2011 ▸; Yamada *et al.*, 2013 ▸). Potential applications of these materials are in catalysis, gas storage (Kitagawa *et al.*, 2004 ▸), luminescence (Allendorf *et al.*, 2015 ▸) or as scintillators (Allendorf *et al.*, 2009 ▸; Doty *et al.*, 2009 ▸; Perry *et al.*, 2012 ▸). Their crystal structures show various non-covalent inter­molecular inter­actions and forces, and therefore are highly connected to their supra­molecular chemistry (Schneider, 2009 ▸) and self-assembly (Cook *et al.*, 2013 ▸). Moreover, these compounds have a high relevance in biological systems inter­acting with macromolecules such as DNA, RNA or proteins (Salonen *et al.*, 2011 ▸), and also in biochemical reactions as protein–ligand recognitions or in drug-delivery systems of biologically active agents (Meyer *et al.*, 2003 ▸). In general, for all these supra­molecular inter­actions, weaker and reversible inter­molecular forces play the key role, including metal coordination, classical and non-classical hydrogen bonding of the types O—H⋯O, N—H⋯O and C—H⋯O, respectively, different π-inter­actions involving the aromatic rings such as π–π stacking, C—H⋯π, ion⋯π and lone-pair⋯π inter­actions. Metal-coordinating and nitro­gen-containing heterocycles such as bi­pyridines and phenanthrolines are electron-deficient aromatic ring systems and thus predestined to be acceptors in π–π stacking, ion⋯π or lone-pair⋯π inter­actions (Janiak, 2000 ▸; Berryman & Johnson, 2009 ▸). In addition, π-donor⋯acceptor functions in different parts of an aromatic mol­ecule can lead to remarkable properties (Albrecht *et al.*, 2010 ▸). Transition-metal coordination compounds with the pseudo aromatic di­amino acid *N*,*N*′-(1,4-phenyl­enedicarbon­yl)diglycine, forming zigzag chains and constructing inter­penetrating networks, have been described in the literature (see *Database survey*).
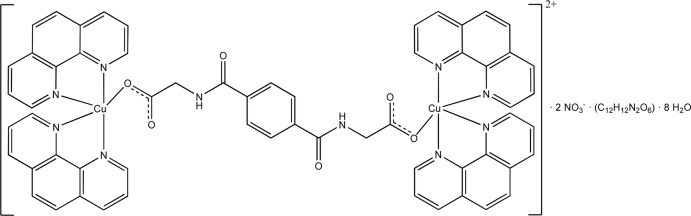


In our synthetic approach, we employ such systems as electron-deficient bidentate aromatic ring systems such as phenanthroline or bi­pyridine in order to block parts of the metal cation coordination sphere. Thus, the alternative assembly process lies in the use of the offered different π-inter­action possibilities, *viz*. π–π stacking, C—H⋯π, ion⋯π and lone-pair⋯π and not in forming the aforementioned zigzag chains. We have previously reported structural studies of two cobalt complexes with bidentate bi­pyridine or bidentate phenanthroline ligands and a non-coordinating *N*,*N*′-(1,4-phenyl­enedicarbon­yl)diglycine mol­ecule in the crystal (Pook *et al.*, 2014 ▸, 2015 ▸). In these structures, the *N*,*N*′-(1,4-phenyl­enedicarbon­yl)diglycine mol­ecule is deprotonated and thus acts as counter-anion. In the two structures, the embedded *N*,*N*′-(1,4-phenyl­enedicarbon­yl)diglycate mol­ecule links the cationic buildings blocks by numerous supra­molecular inter­actions.

In a continuation of this work, we have now synthesized and determined the structure of a novel copper(II) coordination compound where the *N*,*N*′-(1,4-phenyl­enedicarbon­yl)di­glycine moiety is a bis-monodentate bridging anionic ligand in its deprotonated form, as well as a solvent mol­ecule in its neutral form in one crystal structure. The structural investigation and description of the supra­molecular network is confirmed and discussed with the aid of a Hirshfeld surface analysis (Hirshfeld, 1977 ▸; Spackman & Jayatilaka, 2009 ▸) of the cationic complex and the *N*,*N*′-(1,4-phenyl­enedicarbon­yl)diglycine solvent mol­ecule.

## Structural commentary   

**Figure 1:**
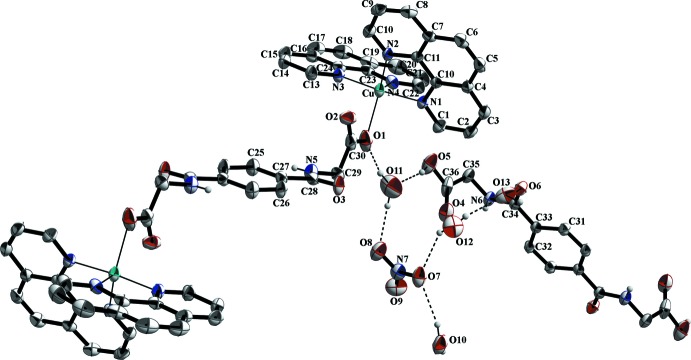
The structures of mol­ecular entities in the title compound with atom labels and displacement ellipsoids of non-H atoms at the 40% probability level. Dashed lines indicate O—H⋯O hydrogen bonds (see Table 1 ▸ for details). Unlabelled atoms are related to labelled ones by the symmetry operation −*x*, −*y*, −*z* + 1 for the cationic complex and −*x*, −*y* + 2, −*z* + 2 for the *N*,*N*′-(1,4-phenyl­enedicarbon­yl)diglycine solvent mol­ecule.

The binuclear and centrosymmetric complex cation of the title compound, [Cu_2_(C_12_H_8_N_2_)_4_(C_12_H_10_N_2_O_6_)](NO_3_)_2_·(C_12_H_12_N_2_O_6_)·8H_2_O, comprises two bidentate phenanthroline ligands and one bridging monodentate *N*,*N*′-(1,4-phenyl­enedicarbon­yl)diglycinate ligand for each Cu^II^ atom, defining a distorted trigonal–bipyramidal coordination sphere. A crystallographic centre of inversion is located at the centroid of the bridging *N*,*N*′-(1,4-phenyl­enedicarbon­yl)diglycinate anion as well as the neutral and non-coordinating *N*,*N*′-(1,4-phenyl­enedicarbon­yl)diglycine solvent mol­ecule. The asymmetric unit is completed by one non-coordinating nitrate counter-anion and four water mol­ecules (Fig. 1 ▸).

The equatorial plane of the Cu^II^ coordination environment is occupied by O1, N2 and N4 atoms with bond lengths of 2.235 (5), 2.119 (2) and 2.111 (3) Å, and the axial positions by N1 and N3 with shorter bonds each of 1.974 (3) Å, respectively. The bond angle N1—Cu—N3 is 174.71 (11)°. The sum of the bond angles O1—Cu—N2 [136.69 (11)°], O1—Cu—N4 [103.88 (12)°] and N2—Cu—N4 [118.90 (10)°] in the equatorial plane amounts to 359.47°, indicating only slight distortions. Distances and angles within the distorted trigonal–bipyramidal coordination sphere of the Cu^II^ ion are similar to those found in the literature (Santha Lakshmi & Samundeeswari, 2015 ▸; Lim *et al.* 2014 ▸). The nearly identical bond lengths of the carboxyl­ate group in the bridging ligand [C30—O1 = 1.249 (5) and C30—O2 = 1.249 (6) Å] indicate a delocalized bonding arrangement, rather than localized single and double bonds as in the case of the carb­oxy­lic group of the neutral *N*,*N*′-(1,4-phenyl­enedicarbon­yl)diglycine solvent mol­ecule [C36—O4 = 1.205 (6) and C36—O5 = 1.316 (5) Å]. The O1—C30—O2 angle of 123.1 (4)° in the carboxyl­ate group is slightly smaller than in the carb­oxy­lic group [O4—C36—O5 = 124.3 (4)°]. In the coordinating *N*,*N*′-(1,4-phenyl­enedicarbon­yl)diglycate ligand, the deviations of atoms defining the central benzamido entity from its least-squares plane are 0.040 (4) Å (C28), −0.084 (3) Å (O3), 0.245 (4) Å (N5) and 0.404 (4) Å (C29), while in the *N*,*N*′-(1,4-phenyl­enedicarbon­yl)diglycine solvent they are −0.018 (4) Å (C34) , 0.102 (3) Å (O6), −0.192 (4) Å (N6) and −0.257 (4) Å (C35). The angle between the amide group and the carboxyl­ate group connected through the *sp* ^3^-hybridized methyl­ene carbon atom (N5—C29—C30) is 113.6 (3)°, and for the neutral solvent mol­ecule it is (N6—C35—C36) 112.1 (3)°. The dihedral angle between the planar carboxyl­ate group (O1/C30/O2) and the aromatic synthon (C25–27/C25′–C27′) of the ligand is 84.1 (3)° and thus smaller than the value found in the free solvent mol­ecule of the aromatic synthon (C31–C33/C31′–C33‘) and the planar carboxyl­ate group (O4/C36/O5) at 88.9 (3)°. The dihedral angle between the mean planes of the two bidentate phenanthroline ligands is 61.71 (5)°; the corresponding value between phenanthroline (N1/C1–C12/N2) and the coordin­ating carboxyl­ate group (O1/C30/O2) is 79.9 (4)° and between phenanthroline (N3/C13–C24/N4) and the carboxyl­ate group is 82.5 (3)°, respectively.

## Supra­molecular features   

**Figure 2:**
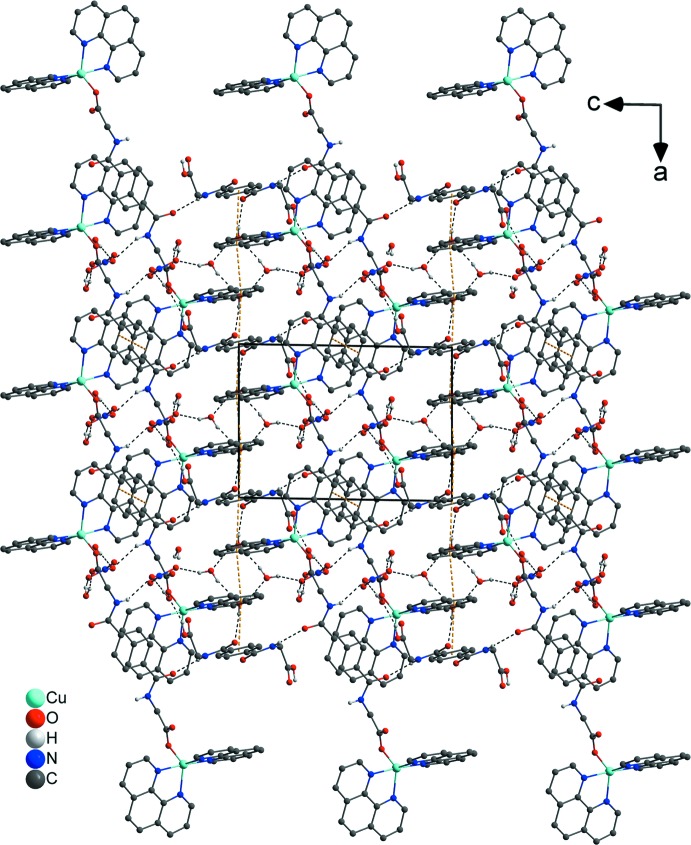
The crystal packing of the title structure in a view along the *b* axis. Selected π–π stacking inter­actions between phenanthroline ligands and *N*,*N*′-(1,4-phenyl­enedicarbon­yl)diglycine solvent and phenanthroline ligands are shown as orange dashed lines as well as classical hydrogen bonding indicated by black dashed lines. The hydrogen atoms of aromatic moieties and methyl­ene groups have been omitted for clarity.

In the crystal structure, numerous non-covalent inter­actions are observed. The nitrate anions are linked *via* O—H⋯O, C—H⋯O and partly *via* N—H⋯O hydrogen bonds with water solvent mol­ecules, the phenanthroline ligands and the metal-coordinating *N*,*N*′-(1,4-phenyl­enedicarbon­yl)di­glycin­ate ligands (Figs. 1 ▸–3 ▸ ▸; Table 1 ▸). π–π inter­actions between parallel-displaced phenanthroline ligands and between phenanthroline and the free *N*,*N*′-(1,4-phenyl­enedicarbon­yl)diglycine solvent mol­ecule, as well as between phenanthroline ligands and the metal-coordinating *N*,*N*′-(1,4-phenyl­enedicarbon­yl)diglycinate ligand stack these components along the different axes (Figs. 2 ▸–4 ▸ ▸). Centroid-to-centroid distances are: 3.6515 (5) Å between *Cg*1⋯*Cg*2, 3.6831 (4) Å between *Cg*2⋯*Cg*4, 3.6686 (5) Å between *Cg*3⋯*Cg*4, and 3.5402 (5) Å between *Cg5*⋯*Cg5*, where *Cg*1, *Cg*2*, *Cg**3*, *Cg**4 and *Cg*5 are the centroids defined by the ring atoms N1/C1–C4/C12, C4–C7/C11–C12, N2/C7–C11, C31–C33/C31′–C33′ and C16–C19/C23–C24, respectively. These distances are in expected ranges (Barceló-Oliver *et al.*, 2010 ▸; Kumar Seth *et al.*, 2010 ▸). In addition, another offset face-to-face arrangement between a phenanthroline and the metal-coordinating *N*,*N*′-(1,4-phenyl­enedicarbon­yl)diglycinate ligand leads to a longer *Cg*6⋯*Cg*7 separation of 4.3673 (4) Å (Fig. 4 ▸), where *Cg*6 and *Cg*7 are the centroids defined by the ring atoms N3/C13–C16/C24 and C25–C27/C25′–C27′, respectively. Such weaker π-stacking inter­actions have been discussed in the past as being relevant (Avasthi *et al.*, 2014 ▸; Dance, 2003 ▸; Janiak, 2000 ▸; Martinez & Iverson, 2012 ▸; Piovesan *et al.*, 2016 ▸; Salonen *et al.*, 2011 ▸). The dihedral angle between the mean planes of the mentioned aromatic rings is 3.50 (12)°. The angle between the lines through C15/*Cg*7 and the centroids through *Cg*6/*Cg*7 is 17.05 (7)° and is slightly increased in comparison with the lines through C26/*Cg*7 and the centroids *Cg*6/*Cg*7 with a value of 16.99 (5)°. Distances shown in Fig. 4 ▸ between atoms and centroids of H15/*Cg*7 and C15/*Cg*7 are 3.4440 (4) and 3.676 (6) Å and between H26/*Cg*7 and C26/*Cg*7 are 3.5049 (4) and 3.713 (5) Å with observed angles of 96.8 (3)° (C15—H15⋯*Cg*7) and 95.4 (3)° (C16—H26⋯*Cg*7), respectively. Besides the previously mentioned forces, a lone-pair⋯π inter­action between the O3 atom of the carboxyl­ate group of the metal-coordinating *N*,*N*′-(1,4-phenyl­enedicarbon­yl)diglycinate ligand and the *Cg*5 centroid of a phenanthroline ligand are observed and associated with a distance of 3.739 (4) Å. This value is similar to those found in the literature (Egli & Sarkhel, 2007 ▸; Gao *et al.*, 2009 ▸; Mooibroek *et al.*, 2008 ▸; Wan *et al.*, 2008 ▸). Finally, π-inter­actions between *Cg*5 and *Cg*7 and the adjacent lone-pair⋯π inter­actions stack the cationic complex subunits along the *a-*axis direction and contribute to the consolidation of the supra­molecular framework (Fig. 4 ▸).

## Hirshfeld surface analysis   

Substanti­ation and visualization of the described supra­molecular features in the crystal structure and their close contacts between different mol­ecular moieties, mol­ecules, ionic and complex subunits can be achieved by using a Hirshfeld surface (HS) analysis (Hirshfeld, 1977 ▸; Spackman & Jayatilaka, 2009 ▸). *Crystal Explorer* (Turner *et al.*, 2017 ▸, Wolff *et al.*, 2012 ▸) offers the possibility to investigate and explore the short atom-to-atom contacts to identify their potential for hydrogen-bonding and π-stacking inter­actions by generating the Hirshfeld surfaces mapped over *d*_norm_, the electrostatic potential, the shape-index and the curvedness.

The HS mapped over *d*_norm_ of the cationic complex subunit in the range −0.7078 to 1.7629 a.u. and of the non-coordinating *N*,*N*′-(1,4-phenyl­enedicarbon­yl)diglycine solvent mol­ecule in the range −0.6806 to 1.9484 a.u. are shown in Fig. 5 ▸ and Fig. 8 ▸, respectively. The corresponding qu­anti­tative contribution of inter­molecular inter­actions are displayed in the overall two-dimensional fingerprint plots (FPs) and those split up into their descending order of crystal cohesion contributions in Fig. 7 ▸ and Fig. 11 ▸, respectively. The white areas of the HS indicate contacts with distances equal to the sum of van der Waals radii and the blue regions indicate longer distances than the van der Waals radii as depicted in Figs. 5 ▸ and 8 ▸. The bright-red spots as indicators of close contacts with shorter distances than the van der Waals radii represent the donor and acceptor functions of dominant classical and non-classical hydrogen-bonding inter­actions of the types O—H⋯O, N—H⋯O and C—H⋯O. This is confirmed by the appearance of large sharp asymmetrical spikes in the H⋯O/O⋯H FPs (Figs. 7 ▸, 11 ▸) in the region of *d*_e_ ∼ 1.19 Å/*d*_i_ ∼ 0.85 Å and *d*_i_ ∼ 0.68 Å/*d*_e_ ∼ 1.02 Å as well as *d*_e_ ∼ 1.05 Å/*d*_i_ ∼ 0.70 Å and *d*_i_ ∼ 1.12 Å/*d*_e_ ∼ 0.78 Å, which comprise 27.9% and 42.2% of the total amount on the HS, respectively.

**Figure 3:**
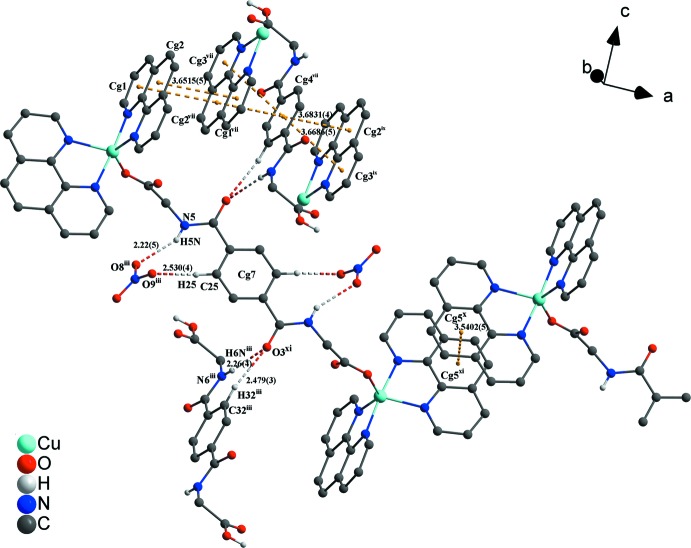
In the crystal packing, different non-covalent inter­actions such as C—H⋯O and N—H⋯O contacts and π–π stacking inter­actions between the aromatic moieties are present. C—H⋯O and N—H⋯O inter­actions are indicated by red–white dashed lines and π–π stacking by dark-yellow dashed lines. The hydrogen atoms not involved in inter­actions have been omitted for clarity. Distances are given in Å. [Symmetry codes: (iii) −*x* + 1, −*y* + 1, −*z* + 1; (vii) −*x* + 1, −*y*, −*z* + 2; (viii) *x* + 1, *y* − 1, *z*; (ix) *x* + 1, *y*, *z*; (x) *x* + 2, *y* − 1, *z*; (xi) −*x* + 2, −*y*, −*z* + 1.]

**Figure 4:**
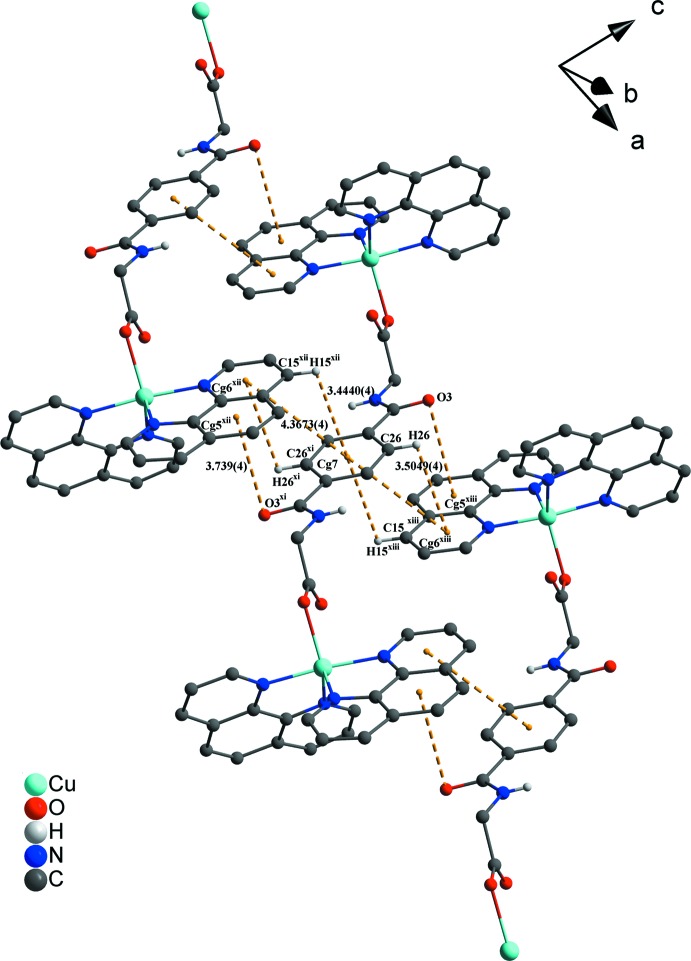
View of the lone-pair⋯π inter­action and π–π stacking between the complex cation subunits. Non-covalent inter­actions are indicated by dark-yellow dashed lines. The hydrogen atoms not involved in inter­actions have been omitted for clarity. Distances are given in Å. [Symmetry codes: (xii) −*x* + 1, −*y*, −*z* + 1; (xiii) *x* + 1, *y*, *z*.]

**Figure 5:**
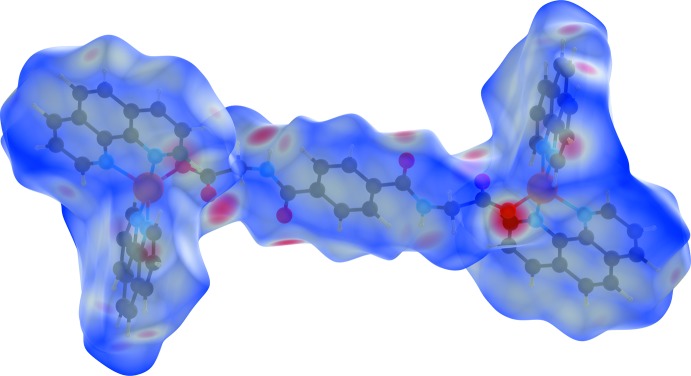
Graphical representation of the three-dimensional Hirshfeld surface (*d*_norm_) for the cationic complex plotted in the range −0.7078 to 1.7629 a.u.. The surface is drawn with transparency and the surface regions with strongest inter­molecular inter­actions are drawn in red.

**Figure 6:**
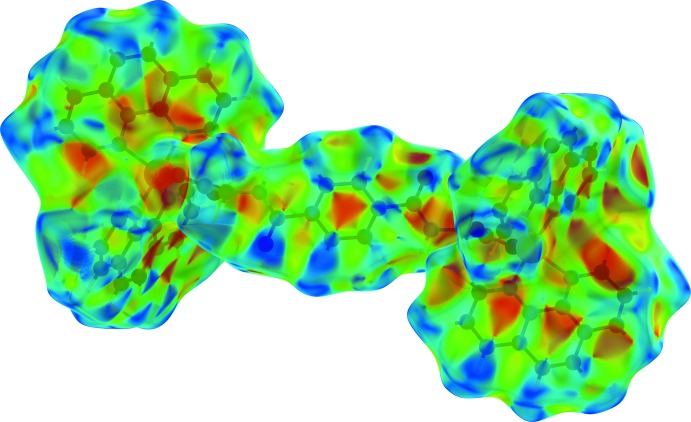
Hirshfeld surface drawn with transparency and mapped over the shape-index for the cationic complex. Red and blue triangles at the phenanthroline ligands and the *N*,*N*′-(1,4-phenyl­enedicarbon­yl)diglycinate ligand indicate the regions involved in π–π stacking inter­actions.

**Figure 7:**
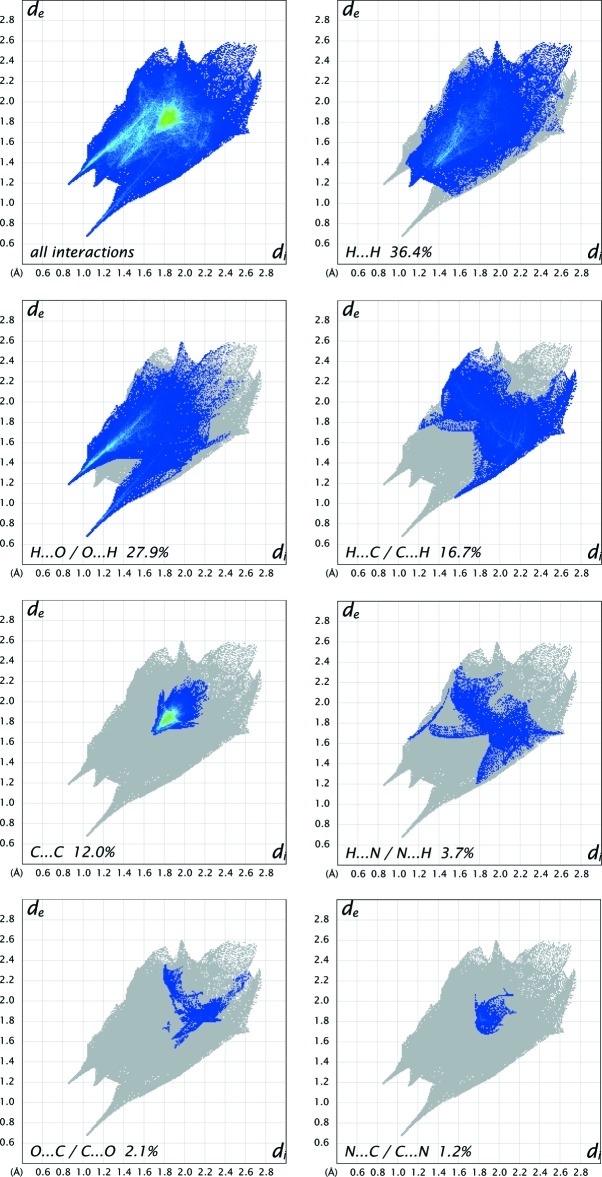
The full two-dimensional fingerprint plots for the cationic-complex subunit, showing all inter­actions and split up into contributions from different contacts. The *d*_i_ and *d*_e_ values are the closest inter­nal and external distances (in Å) from given points on the Hirshfeld surface contacts.

**Figure 8:**
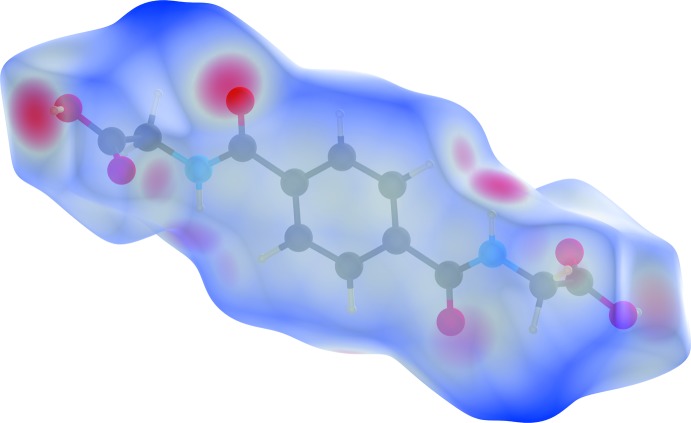
Hirshfeld surface for the *N*,*N*′-(1,4-phenyl­enedicarbon­yl)diglycine solvent mol­ecule with *d*_norm_ over the range −0.6806 to 1.9484 a.u.. The surface is drawn with transparency; red spots indicate the strongest inter­molecular inter­actions.

**Figure 10:**
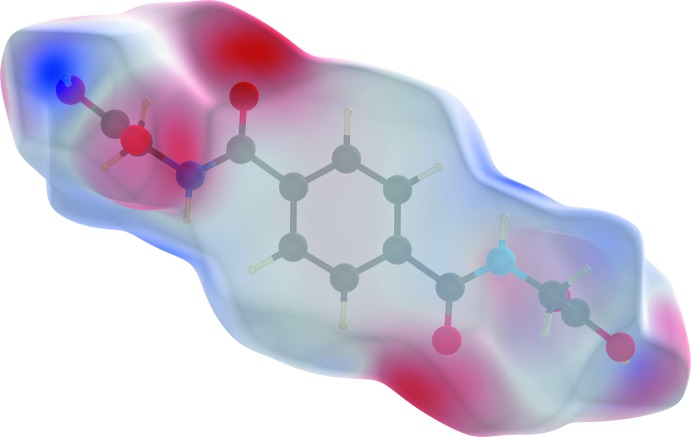
View of the transparent three-dimensional Hirshfeld surface of the *N*,*N*′-(1,4-phenyl­enedicarbon­yl)diglycine solvent mol­ecule plotted over electrostatic potential energy in the range −0.0828 to 0.1815 a.u. using the STO-3G basis set at the Hartree–Fock level. The O—H⋯O, N—H⋯O and C—H⋯O hydrogen-bond donor and acceptor atoms are displayed as blue and red regions around the atoms corresponding to positive and negative potentials, respectively.

**Figure 11:**
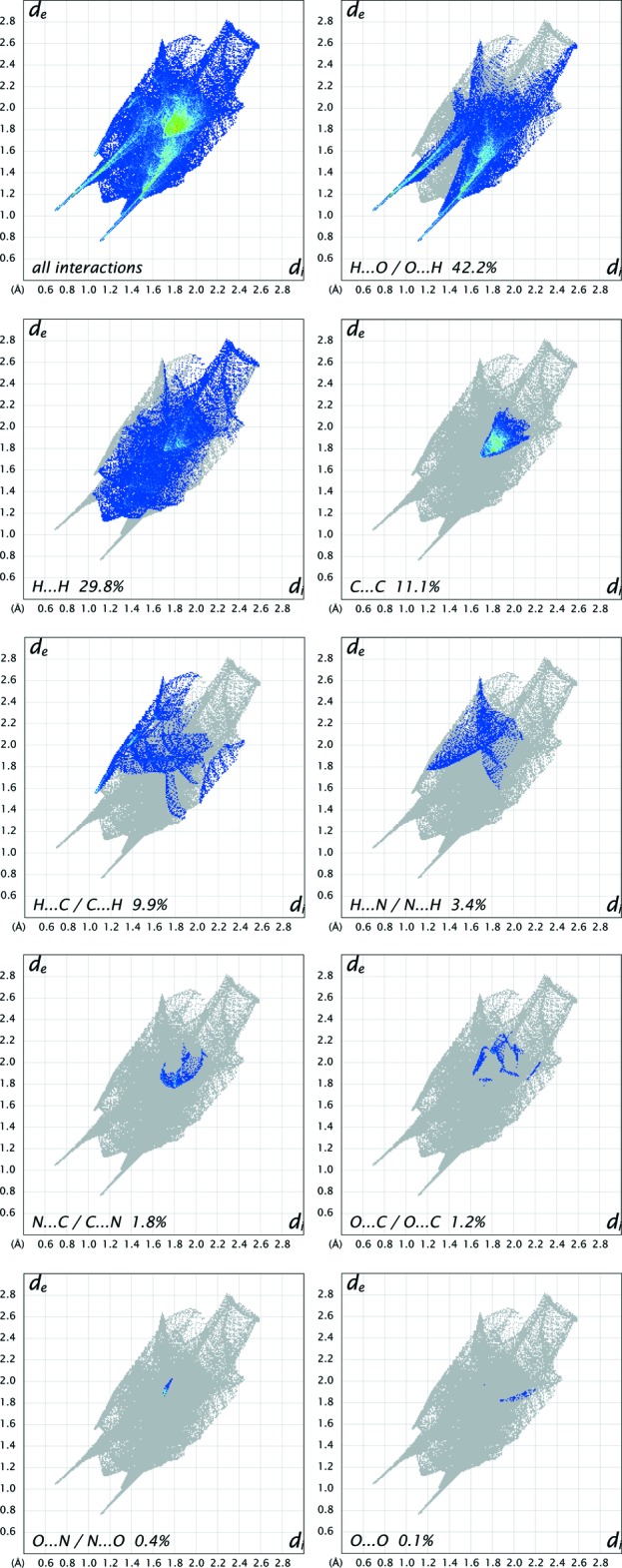
The full two-dimensional fingerprint plots for the *N*,*N*′-(1,4-phenyl­enedicarbon­yl)diglycine solvent mol­ecule, showing all inter­actions, and delineated into the contributions from different contacts. The *d*_i_ and *d*_e_ values are the closest inter­nal and external distances (in Å) from given points on the Hirshfeld surface contacts.

In order to classify the donor and acceptor groups of the *N*,*N*′-(1,4-phenyl­enedicarbon­yl)diglycine solvent mol­ecule involved in hydrogen bonding, the HS mapped over the electrostatic potentials were calculated using *TONTO* (Spackman *et al.*, 2008 ▸; Jayatilaka *et al.*, 2005 ▸) with standard settings of the STP-3G basis set at Hartree–Fock theory. The appearance of blue and red surface regions indicates the positive and negative electrostatic potential as shown in Fig. 10 ▸, suggesting that the carbonyl oxygen atom of the amide group and the non-protonated oxygen atom of the carboxyl­ate group act as hydrogen-bond acceptors whereas the nitro­gen/hydrogen atoms of the amide group and the protonated oxygen atom of the carb­oxy group as well as the carbon/hydrogen atoms of the aromatic moiety act as hydrogen-bond donors. H⋯H contacts compromise 36.4% to the cationic complex as the largest contribution and 29.8% to the *N*,*N*′-(1,4-phenyl­enedicarbon­yl)diglycine solvent mol­ecule as the second largest contribution within the HS. This high relevance for the HS is attributed to the high proportion of hydrogen atoms in the structure of these entities. The H⋯N/N⋯H contacts contribute 3.7% to the cationic complex and 3.4% to the solvent mol­ecule to the total HS, respectively. Short contacts of the solvent mol­ecule with a minor contribution to the lattice of O⋯N/N⋯O (0.4%) and O⋯O (0.1%) are also observed. The contribution of the different π–π inter­actions used for the stacking of the cationic complex subunits and the *N*,*N*′-(1,4-phenyl­enedicarbon­yl)diglycine solvent mol­ecule along the different axes is also significant for both entities. Therefore, the close H⋯C/C⋯H (16.7%), C⋯C (12.0%), O⋯C/C⋯O (2.1%) and N⋯C/C⋯N (1.2%) contacts of the cationic complex are assigned to C—H⋯π inter­actions, π–π stacking (face-to-face) and lone-pair⋯π inter­actions of the carbonyl group and stacking between the phenanthroline ligands (Figs. 3 ▸, 4 ▸ and 7 ▸). For the *N*,*N*′-(1,4-phenyl­enedicarbon­yl)diglycine solvent mol­ecule, the close C⋯C (11.1%), H⋯C/C⋯H (9.9%), N⋯C/C⋯N (1.8%) and O⋯C/C⋯O (1.2%) contacts are assigned to π–π stacking (face-to-face), C—H⋯π inter­actions and stackings of the phenanthrolines and lone-pair⋯π inter­actions of the carbonyl group (Figs. 3 ▸, 4 ▸ and 11 ▸). This corresponds to the appearance of the red triangles of the aromatic moieties of the bidentate phenanthroline ligands, the metal-coordinating *N*,*N*′-(1,4-phenyl­enedicarbon­yl)diglycinate ligand as well as the non-coordinating *N*,*N*′-(1,4-phenyl­enedicarbon­yl)di­glycine solvent mol­ecule in the HS mapped over the shape-index, which represent the face-to-face π–π stacking inter­actions (Figs. 6 ▸, 9 ▸).

## Database survey   

A search for crystal structures containing the ligand *N*,*N*′-(1,4-phenyl­enedicarbon­yl)diglycine using *SciFinder* (SciFinder, 2019 ▸) and the Cambridge Structural Database (Version 5.35, November 2013 with three updates; Groom *et al.*, 2016 ▸) resulted in six entries (Duan *et al.*, 2010 ▸; Kostakis *et al.*, 2005 ▸, 2011 ▸; Zhang *et al.*, 2005 ▸, 2006 ▸). Some of these structures are composed of inter­penetrating networks as mentioned in the *Chemical context*. Among them are two structures which include bi­pyridine or phenanthroline ligands besides *N*,*N*′-(1,4-phenyl­enedicarbon­yl)diglycine, and their structures show a number of non-classical inter­actions (Pook *et al.*, 2014 ▸, 2015 ▸).

## Synthesis and crystallization   

The starting material, *N*,*N*′-(1,4-phenyl­enedicarbon­yl) diglycine, was prepared by the method of Cleaver & Pratt (1955 ▸). Cesium carbonate (2 mmol), 1,10-phenanthroline (1 mmol) and 2,2′-(benzene-1,4-dicarboxamido)­diacetatic acid (1 mmol) were dissolved in a 1:1 (*v*/*v*) mixture of water and methanol (50 ml) and refluxed for 30 minutes. The mixture was allowed to cool to room temperature, and a previously prepared aqueous solution of copper acetate (1 mmol) was slowly added under continuous stirring. Pale-blue block-shaped crystals of the title compound were obtained by slow evaporation at room temperature.

## Refinement   

Crystal data, data collection and structure refinement details are summarized in Table 2 ▸. All C-bound H atoms were positioned with idealized geometry and refined with *U*_iso_(H) = 1.2*U*_eq_(C) and C—H(aromatic) = 0.94 Å and C—H(methyl­ene) = 0.98 Å using a riding model. The water H atoms were located in a difference-Fourier map and were refined with O—H distances restrained to 0.82–0.87 Å and with *U*_iso_(H) = 1.5*U*_eq_(O), except O11—H11*A* with a fixed distance of 1.00 Å, which led to a stable and consolidated hydrogen-bonding network.

## Supplementary Material

Crystal structure: contains datablock(s) I. DOI: 10.1107/S2056989019005164/wm5501sup1.cif

Structure factors: contains datablock(s) I. DOI: 10.1107/S2056989019005164/wm5501Isup2.hkl

Supporting information file. DOI: 10.1107/S2056989019005164/wm5501Isup3.cdx

CCDC reference: 1910262

Additional supporting information:  crystallographic information; 3D view; checkCIF report

## Figures and Tables

**Figure 9:**
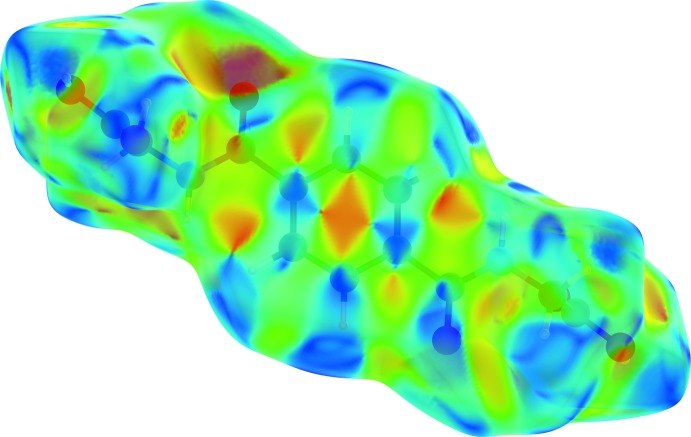
Hirshfeld surfaces drawn with transparency and mapped over the shape-index for the *N*,*N*′-(1,4-phenyl­enedicarbon­yl)diglycine solvent mol­ecule. Red and blue triangles indicate the region involved in π–π stacking inter­actions.

**Table 1:** Hydrogen-bond geometry (Å, °)

*D*—H⋯*A*	*D*—H	H⋯*A*	*D*⋯*A*	*D*—H⋯*A*
O5—H5⋯O11	0.87 (6)	1.84 (6)	2.665 (5)	159 (6)
O10—H10*A*⋯O2^i^	0.83 (7)	2.06 (7)	2.878 (6)	168 (7)
O10—H10*B*⋯O7	0.85 (7)	2.24 (7)	3.033 (6)	155 (7)
O11—H11*A*⋯O8	0.85	2.00	2.848 (6)	179
O11—H11*B*⋯O1	1.00	1.68	2.643 (6)	160
O12—H12*A*⋯O7	0.79 (8)	2.32 (8)	3.024 (6)	149 (8)
O12—H12*B*⋯O13	0.80 (8)	2.07 (8)	2.856 (6)	170 (8)
O13—H13*A*⋯O12^ii^	0.75 (7)	2.05 (7)	2.757 (6)	157 (8)
O13—H13*B*⋯O6	0.81 (7)	2.05 (7)	2.851 (4)	170 (7)
N5—H5*N*⋯O8^iii^	0.79 (4)	2.23 (5)	2.947 (4)	152 (4)
N6—H6⋯O3^iv^	0.75 (4)	2.26 (4)	2.995 (4)	167 (4)
C2—H2⋯O13	0.94	2.37	3.302 (5)	172
C3—H3⋯O10^ii^	0.94	2.44	3.358 (7)	165
C9—H9⋯O4^v^	0.94	2.40	3.099 (5)	131
C13—H13⋯O2	0.94	2.42	3.031 (5)	122
C14—H14⋯O10^iii^	0.94	2.60	3.414 (5)	145
C25—H25⋯O9^iii^	0.94	2.53	3.354 (6)	147
C32—H32⋯O3^iv^	0.94	2.48	3.388 (4)	163
C35—H35*A*⋯O10^vi^	0.98	2.53	3.378 (5)	145

Symmetry codes: (i) 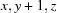; (ii) 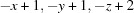; (iii) 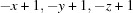; (iv) 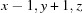; (v) 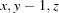; (vi) 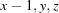.

**Table 2:** Experimental details

Crystal data	
Chemical formula	[Cu_2_(C_12_H_10_N_2_O_6_)(C_12_H_8_N_2_)_4_](NO_3_)_2_·C_12_H_12_N_2_O_6_·8H_2_O
*M*_r_	1674.50
Crystal system, space group	Triclinic, *P* 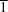
Temperature (K)	223
*a*, *b*, *c* (Å)	11.0448 (12), 13.0793 (15), 15.419 (2)
α, β, γ (°)	65.322 (10), 81.013 (11), 66.007 (8)
*V* (Å^3^)	1848.8 (4)
*Z*	1
Radiation type	Mo *K*α
μ (mm^−1^)	0.67
Crystal size (mm)	0.25 × 0.23 × 0.21

Data collection	
Diffractometer	Stoe *IPDS* 2
Absorption correction	Numerical (*X-AREA, *X-RED32**; Stoe, 2008 ▸)
*T*_min_, *T*_max_	0.768, 0.791
No. of measured, independent and observed [*I* > 2σ(*I*)] reflections	22995, 6493, 5435
*R*_int_	0.047
(sin θ/λ)_max_ (Å^−1^)	0.595

Refinement	
*R*[*F* ^2^ > 2σ(*F* ^2^)], *wR*(*F* ^2^), *S*	0.052, 0.129, 1.04
No. of reflections	6493
No. of parameters	542
H-atom treatment	H atoms treated by a mixture of independent and constrained refinement
Δρ_max_, Δρ_min_ (e Å^−3^)	1.33, −0.70

Computer programs: *X-AREA* and *X-RED* (Stoe, 2008 ▸), *SHELXS97* (Sheldrick, 2008 ▸), *SHELXL2018* (Sheldrick, 2015 ▸), *DIAMOND* (Brandenburg 2007 ▸), *PLATON* (Spek, 2009 ▸) and *publCIF* (Westrip, 2010 ▸).

## supplementary crystallographic information

### Crystal data

**Table:**

[Cu_2_(C_12_H_10_N_2_O_6_)(C_12_H_8_N_2_)_4_](NO_3_)_2_·C_12_H_12_N_2_O_6_·8H_2_O	*Z* = 1
*M**_r_* = 1674.50	*F*(000) = 866
Triclinic, *P*1	*D*_x_ = 1.504 Mg m^−^^3^
*a* = 11.0448 (12) Å	Mo *K*α radiation, λ = 0.71073 Å
*b* = 13.0793 (15) Å	Cell parameters from 25382 reflections
*c* = 15.419 (2) Å	θ = 4.0–62.1°
α = 65.322 (10)°	µ = 0.67 mm^−^^1^
β = 81.013 (11)°	*T* = 223 K
γ = 66.007 (8)°	Block, blue
*V* = 1848.8 (4) Å^3^	0.25 × 0.23 × 0.21 mm

### Data collection

**Table:**

Stoe IPDS 2 diffractometer	6493 independent reflections
Radiation source: sealed X-ray tube, 12 x 0.4 mm long-fine focus	5435 reflections with *I* > 2σ(*I*)
Plane graphite monochromator	*R*_int_ = 0.047
Detector resolution: 6.67 pixels mm^-1^	θ_max_ = 25.0°, θ_min_ = 2.0°
rotation method scans	*h* = −13→13
Absorption correction: numerical (*X-AREA, X-RED32*; Stoe, 2008)	*k* = −15→15
*T*_min_ = 0.768, *T*_max_ = 0.791	*l* = −18→18
22995 measured reflections	

### Refinement

**Table:**

Refinement on *F*^2^	0 restraints
Least-squares matrix: full	Hydrogen site location: mixed
*R*[*F*^2^ > 2σ(*F*^2^)] = 0.052	H atoms treated by a mixture of independent and constrained refinement
*wR*(*F*^2^) = 0.129	*w* = 1/[σ^2^(*F*_o_^2^) + (0.058*P*)^2^ + 2.4056*P*] where *P* = (*F*_o_^2^ + 2*F*_c_^2^)/3
*S* = 1.04	(Δ/σ)_max_ = 0.001
6493 reflections	Δρ_max_ = 1.33 e Å^−^^3^
542 parameters	Δρ_min_ = −0.70 e Å^−^^3^

### Special details

**Table:**

Geometry. All esds (except the esd in the dihedral angle between two l.s. planes) are estimated using the full covariance matrix. The cell esds are taken into account individually in the estimation of esds in distances, angles and torsion angles; correlations between esds in cell parameters are only used when they are defined by crystal symmetry. An approximate (isotropic) treatment of cell esds is used for estimating esds involving l.s. planes.

### Fractional atomic coordinates and isotropic or equivalent isotropic displacement parameters (Å^2^)

**Table:**

	*x*	*y*	*z*	*U*_iso_*/*U*_eq_	
Cu	0.25718 (4)	0.21545 (4)	0.74008 (3)	0.03391 (13)	
O1	0.3776 (3)	0.3298 (4)	0.6699 (2)	0.0753 (10)	
O2	0.4964 (4)	0.1408 (4)	0.6874 (2)	0.0757 (10)	
O3	0.8457 (3)	0.1118 (2)	0.69057 (17)	0.0501 (6)	
O4	0.1969 (3)	0.7417 (3)	0.7625 (2)	0.0674 (9)	
O5	0.1003 (4)	0.6311 (3)	0.7449 (3)	0.0756 (10)	
H5	0.184 (6)	0.598 (6)	0.735 (4)	0.10 (2)*	
O6	0.0556 (3)	0.7144 (2)	0.98070 (18)	0.0470 (6)	
O7	0.5373 (3)	0.7260 (3)	0.7046 (2)	0.0658 (8)	
O8	0.4738 (3)	0.7032 (3)	0.5920 (3)	0.0721 (9)	
O9	0.4346 (3)	0.8821 (3)	0.5846 (3)	0.0763 (10)	
O10	0.6564 (4)	0.8916 (4)	0.7169 (3)	0.0788 (11)	
H10A	0.609 (7)	0.965 (7)	0.700 (5)	0.118*	
H10B	0.601 (7)	0.860 (6)	0.720 (5)	0.118*	
O11	0.3366 (4)	0.5419 (4)	0.6730 (4)	0.1124 (16)	
H11A	0.377732	0.589890	0.649309	0.169*	
H11B	0.372532	0.460190	0.669708	0.169*	
O12	0.5209 (4)	0.5019 (4)	0.8692 (3)	0.0860 (12)	
H12A	0.509 (8)	0.552 (7)	0.817 (6)	0.129*	
H12B	0.456 (8)	0.509 (7)	0.901 (6)	0.129*	
O13	0.3103 (4)	0.5241 (3)	1.0022 (3)	0.0735 (10)	
H13A	0.340 (7)	0.514 (7)	1.047 (5)	0.110*	
H13B	0.242 (7)	0.580 (6)	1.001 (5)	0.110*	
N1	0.2860 (3)	0.2114 (2)	0.86480 (19)	0.0344 (6)	
N2	0.2949 (2)	0.0293 (2)	0.82400 (18)	0.0316 (6)	
N3	0.2122 (3)	0.2190 (3)	0.61963 (19)	0.0374 (6)	
N4	0.0604 (3)	0.3436 (3)	0.7252 (2)	0.0383 (6)	
N5	0.6970 (3)	0.2107 (3)	0.5731 (2)	0.0442 (7)	
H5N	0.676 (4)	0.222 (4)	0.522 (3)	0.053*	
N6	−0.0342 (3)	0.8618 (3)	0.8420 (2)	0.0403 (7)	
H6	−0.060 (4)	0.928 (4)	0.809 (3)	0.048*	
N7	0.4814 (3)	0.7715 (3)	0.6274 (3)	0.0527 (8)	
C1	0.2822 (4)	0.3037 (3)	0.8825 (3)	0.0443 (8)	
H1	0.267837	0.379334	0.831627	0.053*	
C2	0.2987 (4)	0.2912 (4)	0.9743 (3)	0.0495 (9)	
H2	0.293266	0.358439	0.985341	0.059*	
C3	0.3229 (3)	0.1814 (4)	1.0482 (3)	0.0452 (9)	
H3	0.334490	0.172589	1.110445	0.054*	
C4	0.3306 (3)	0.0807 (3)	1.0318 (2)	0.0356 (7)	
C5	0.3589 (3)	−0.0393 (4)	1.1040 (2)	0.0447 (9)	
H5C	0.371489	−0.053382	1.167503	0.054*	
C6	0.3681 (3)	−0.1326 (4)	1.0830 (3)	0.0453 (9)	
H6C	0.387433	−0.210433	1.131931	0.054*	
C7	0.3489 (3)	−0.1146 (3)	0.9879 (2)	0.0362 (7)	
C8	0.3598 (3)	−0.2085 (3)	0.9613 (3)	0.0446 (8)	
H8	0.381504	−0.288631	1.007015	0.054*	
C9	0.3387 (3)	−0.1819 (3)	0.8691 (3)	0.0457 (9)	
H9	0.346209	−0.243619	0.849851	0.055*	
C10	0.3055 (3)	−0.0621 (3)	0.8023 (3)	0.0384 (7)	
H10	0.289800	−0.045355	0.738683	0.046*	
C11	0.3174 (3)	0.0027 (3)	0.9160 (2)	0.0291 (6)	
C12	0.3098 (3)	0.1013 (3)	0.9378 (2)	0.0303 (6)	
C13	0.2907 (4)	0.1582 (4)	0.5673 (3)	0.0443 (8)	
H13	0.378778	0.107489	0.587836	0.053*	
C14	0.2470 (4)	0.1674 (4)	0.4843 (3)	0.0505 (9)	
H14	0.304078	0.123004	0.449269	0.061*	
C15	0.1214 (4)	0.2409 (4)	0.4545 (3)	0.0539 (10)	
H15	0.090157	0.247967	0.398148	0.065*	
C16	0.0357 (4)	0.3078 (3)	0.5073 (3)	0.0460 (9)	
C17	−0.0983 (4)	0.3896 (4)	0.4813 (3)	0.0599 (11)	
H17	−0.134768	0.400995	0.425248	0.072*	
C18	−0.1743 (4)	0.4512 (4)	0.5349 (3)	0.0597 (12)	
H18	−0.262002	0.504503	0.514847	0.072*	
C19	−0.1264 (3)	0.4381 (3)	0.6200 (3)	0.0489 (9)	
C20	−0.2005 (4)	0.5012 (4)	0.6777 (4)	0.0607 (12)	
H20	−0.289424	0.553980	0.661767	0.073*	
C21	−0.1440 (4)	0.4859 (4)	0.7556 (3)	0.0601 (11)	
H21	−0.191930	0.528552	0.794160	0.072*	
C22	−0.0130 (4)	0.4056 (3)	0.7777 (3)	0.0486 (9)	
H22	0.025920	0.394412	0.832696	0.058*	
C23	0.0045 (3)	0.3590 (3)	0.6474 (2)	0.0370 (7)	
C24	0.0857 (3)	0.2931 (3)	0.5906 (2)	0.0364 (7)	
C25	0.8721 (3)	0.0698 (4)	0.4687 (3)	0.0458 (9)	
H25	0.784492	0.117261	0.445384	0.055*	
C26	1.0356 (3)	−0.0078 (4)	0.5836 (2)	0.0450 (9)	
H26	1.062733	−0.014694	0.641321	0.054*	
C27	0.9071 (3)	0.0624 (3)	0.5529 (2)	0.0341 (7)	
C28	0.8138 (3)	0.1297 (3)	0.6120 (2)	0.0369 (7)	
C29	0.6000 (3)	0.2888 (3)	0.6167 (3)	0.0441 (8)	
H29A	0.565464	0.372051	0.568373	0.053*	
H29B	0.644011	0.289430	0.666847	0.053*	
C30	0.4828 (4)	0.2492 (5)	0.6605 (3)	0.0528 (10)	
C31	0.0363 (3)	0.8779 (3)	1.0573 (2)	0.0357 (7)	
H31	0.061328	0.794751	1.096732	0.043*	
C32	−0.0312 (3)	1.0411 (3)	0.9051 (2)	0.0357 (7)	
H32	−0.052282	1.069462	0.840293	0.043*	
C33	0.0048 (3)	0.9183 (3)	0.9624 (2)	0.0318 (7)	
C34	0.0115 (3)	0.8235 (3)	0.9290 (2)	0.0353 (7)	
C35	−0.0341 (3)	0.7756 (4)	0.8067 (3)	0.0461 (9)	
H35A	−0.100359	0.817367	0.755127	0.055*	
H35B	−0.059411	0.712505	0.858186	0.055*	
C36	0.1019 (4)	0.7156 (4)	0.7694 (3)	0.0563 (11)	

### Atomic displacement parameters (Å^2^)

**Table:**

	*U*^11^	*U*^22^	*U*^33^	*U*^12^	*U*^13^	*U*^23^
Cu	0.0328 (2)	0.0360 (2)	0.0307 (2)	−0.00752 (16)	−0.00332 (15)	−0.01511 (17)
O1	0.0422 (16)	0.131 (3)	0.0574 (18)	−0.0224 (18)	0.0062 (13)	−0.053 (2)
O2	0.106 (3)	0.099 (3)	0.0428 (16)	−0.075 (2)	−0.0118 (16)	−0.0072 (17)
O3	0.0499 (14)	0.0586 (17)	0.0344 (13)	−0.0057 (12)	−0.0082 (11)	−0.0230 (12)
O4	0.0387 (15)	0.101 (3)	0.0662 (19)	−0.0118 (15)	−0.0036 (13)	−0.0495 (19)
O5	0.069 (2)	0.071 (2)	0.093 (3)	−0.0012 (17)	−0.0093 (18)	−0.059 (2)
O6	0.0528 (14)	0.0393 (15)	0.0485 (15)	−0.0111 (12)	−0.0088 (12)	−0.0197 (12)
O7	0.0598 (17)	0.074 (2)	0.0545 (18)	−0.0071 (15)	−0.0100 (14)	−0.0307 (16)
O8	0.0640 (19)	0.081 (2)	0.083 (2)	−0.0173 (17)	−0.0116 (16)	−0.048 (2)
O9	0.0559 (18)	0.056 (2)	0.097 (3)	−0.0071 (15)	−0.0173 (17)	−0.0196 (19)
O10	0.072 (2)	0.114 (3)	0.066 (2)	−0.031 (2)	−0.0087 (17)	−0.051 (2)
O11	0.102 (3)	0.073 (3)	0.145 (4)	−0.017 (2)	0.042 (3)	−0.056 (3)
O12	0.080 (2)	0.066 (2)	0.082 (3)	−0.008 (2)	−0.017 (2)	−0.015 (2)
O13	0.062 (2)	0.0505 (19)	0.109 (3)	−0.0093 (15)	−0.0154 (19)	−0.038 (2)
N1	0.0352 (14)	0.0343 (15)	0.0353 (14)	−0.0101 (11)	−0.0006 (11)	−0.0177 (12)
N2	0.0283 (12)	0.0362 (15)	0.0320 (13)	−0.0099 (11)	−0.0002 (10)	−0.0168 (12)
N3	0.0344 (14)	0.0431 (17)	0.0327 (14)	−0.0112 (12)	−0.0031 (11)	−0.0151 (13)
N4	0.0328 (14)	0.0353 (15)	0.0381 (15)	−0.0087 (12)	0.0021 (12)	−0.0112 (12)
N5	0.0367 (15)	0.0496 (19)	0.0401 (16)	−0.0054 (13)	−0.0054 (13)	−0.0206 (15)
N6	0.0386 (15)	0.0457 (18)	0.0406 (17)	−0.0099 (14)	−0.0050 (12)	−0.0247 (14)
N7	0.0346 (15)	0.056 (2)	0.063 (2)	−0.0049 (15)	−0.0018 (15)	−0.0294 (19)
C1	0.051 (2)	0.039 (2)	0.049 (2)	−0.0165 (16)	0.0015 (16)	−0.0242 (17)
C2	0.048 (2)	0.057 (2)	0.063 (2)	−0.0208 (18)	0.0056 (18)	−0.043 (2)
C3	0.0381 (18)	0.068 (3)	0.045 (2)	−0.0213 (17)	0.0056 (15)	−0.038 (2)
C4	0.0261 (14)	0.054 (2)	0.0323 (16)	−0.0158 (14)	0.0006 (12)	−0.0208 (15)
C5	0.0358 (17)	0.067 (3)	0.0296 (17)	−0.0229 (17)	−0.0025 (13)	−0.0126 (17)
C6	0.0395 (18)	0.049 (2)	0.0352 (18)	−0.0175 (16)	−0.0076 (14)	−0.0023 (16)
C7	0.0288 (15)	0.0389 (19)	0.0378 (17)	−0.0143 (14)	−0.0017 (13)	−0.0103 (15)
C8	0.0388 (18)	0.0356 (19)	0.054 (2)	−0.0140 (15)	−0.0057 (16)	−0.0108 (17)
C9	0.0405 (18)	0.041 (2)	0.065 (2)	−0.0160 (16)	0.0023 (17)	−0.0298 (19)
C10	0.0352 (17)	0.044 (2)	0.0425 (18)	−0.0116 (14)	−0.0011 (14)	−0.0250 (16)
C11	0.0210 (13)	0.0348 (17)	0.0315 (15)	−0.0098 (12)	−0.0009 (11)	−0.0133 (13)
C12	0.0239 (14)	0.0376 (18)	0.0311 (16)	−0.0119 (13)	0.0010 (12)	−0.0150 (14)
C13	0.0409 (18)	0.054 (2)	0.0412 (19)	−0.0151 (16)	−0.0017 (15)	−0.0237 (17)
C14	0.060 (2)	0.067 (3)	0.0381 (19)	−0.031 (2)	0.0043 (17)	−0.0273 (19)
C15	0.069 (3)	0.066 (3)	0.0359 (19)	−0.038 (2)	−0.0098 (18)	−0.0132 (18)
C16	0.050 (2)	0.047 (2)	0.0390 (19)	−0.0273 (17)	−0.0134 (16)	−0.0024 (16)
C17	0.058 (2)	0.059 (3)	0.054 (2)	−0.028 (2)	−0.025 (2)	0.000 (2)
C18	0.043 (2)	0.049 (2)	0.069 (3)	−0.0169 (18)	−0.023 (2)	0.001 (2)
C19	0.0328 (17)	0.035 (2)	0.060 (2)	−0.0125 (15)	−0.0055 (16)	0.0001 (17)
C20	0.0341 (19)	0.044 (2)	0.082 (3)	−0.0043 (17)	−0.002 (2)	−0.013 (2)
C21	0.046 (2)	0.046 (2)	0.072 (3)	−0.0066 (18)	0.016 (2)	−0.026 (2)
C22	0.046 (2)	0.043 (2)	0.048 (2)	−0.0110 (16)	0.0069 (16)	−0.0168 (17)
C23	0.0332 (16)	0.0327 (18)	0.0368 (17)	−0.0137 (14)	−0.0030 (13)	−0.0037 (14)
C24	0.0364 (17)	0.0355 (18)	0.0326 (16)	−0.0170 (14)	−0.0055 (13)	−0.0038 (14)
C25	0.0313 (16)	0.060 (2)	0.0398 (19)	−0.0064 (16)	−0.0079 (14)	−0.0220 (18)
C26	0.0366 (17)	0.062 (2)	0.0346 (18)	−0.0093 (16)	−0.0069 (14)	−0.0236 (17)
C27	0.0332 (16)	0.0371 (18)	0.0292 (16)	−0.0132 (14)	−0.0012 (12)	−0.0100 (14)
C28	0.0350 (17)	0.0406 (19)	0.0335 (17)	−0.0163 (14)	0.0012 (13)	−0.0114 (15)
C29	0.0357 (17)	0.046 (2)	0.047 (2)	−0.0123 (15)	0.0040 (15)	−0.0196 (17)
C30	0.045 (2)	0.085 (3)	0.0306 (18)	−0.023 (2)	−0.0071 (15)	−0.023 (2)
C31	0.0375 (16)	0.0348 (18)	0.0329 (16)	−0.0127 (14)	−0.0050 (13)	−0.0108 (14)
C32	0.0369 (16)	0.0442 (19)	0.0272 (15)	−0.0142 (14)	−0.0027 (13)	−0.0152 (14)
C33	0.0240 (14)	0.0397 (18)	0.0334 (16)	−0.0095 (13)	0.0008 (12)	−0.0186 (14)
C34	0.0286 (15)	0.042 (2)	0.0384 (18)	−0.0111 (14)	0.0015 (13)	−0.0214 (16)
C35	0.0428 (19)	0.056 (2)	0.052 (2)	−0.0147 (17)	−0.0043 (16)	−0.0344 (19)
C36	0.062 (3)	0.054 (2)	0.040 (2)	0.005 (2)	−0.0153 (18)	−0.0273 (19)

### Geometric parameters (Å, º)

**Table:**

Cu—N1	1.974 (3)	C6—H6C	0.9400
Cu—N3	1.974 (3)	C7—C11	1.404 (5)
Cu—N4	2.111 (3)	C7—C8	1.406 (5)
Cu—N2	2.119 (3)	C8—C9	1.350 (5)
Cu—O1	2.235 (4)	C8—H8	0.9400
O1—C30	1.249 (5)	C9—C10	1.397 (5)
O2—C30	1.249 (6)	C9—H9	0.9400
O3—C28	1.214 (4)	C10—H10	0.9400
O4—C36	1.205 (6)	C11—C12	1.431 (4)
O5—C36	1.316 (5)	C13—C14	1.379 (5)
O5—H5	0.87 (6)	C13—H13	0.9400
O6—C34	1.231 (4)	C14—C15	1.344 (6)
O7—N7	1.221 (4)	C14—H14	0.9400
O8—N7	1.259 (5)	C15—C16	1.415 (6)
O9—N7	1.224 (5)	C15—H15	0.9400
O10—H10A	0.83 (7)	C16—C24	1.387 (5)
O10—H10B	0.85 (7)	C16—C17	1.426 (6)
O11—H11A	0.8498	C17—C18	1.352 (7)
O11—H11B	0.9981	C17—H17	0.9400
O12—H12A	0.79 (8)	C18—C19	1.408 (6)
O12—H12B	0.80 (8)	C18—H18	0.9400
O13—H13A	0.75 (7)	C19—C23	1.395 (5)
O13—H13B	0.81 (7)	C19—C20	1.408 (6)
N1—C1	1.328 (4)	C20—C21	1.343 (7)
N1—C12	1.357 (4)	C20—H20	0.9400
N2—C10	1.328 (4)	C21—C22	1.390 (6)
N2—C11	1.350 (4)	C21—H21	0.9400
N3—C13	1.338 (5)	C22—H22	0.9400
N3—C24	1.350 (4)	C23—C24	1.439 (5)
N4—C22	1.334 (5)	C25—C27	1.366 (5)
N4—C23	1.338 (4)	C25—C26^i^	1.401 (5)
N5—C28	1.322 (4)	C25—H25	0.9400
N5—C29	1.450 (5)	C26—C27	1.368 (5)
N5—H5N	0.79 (4)	C26—H26	0.9400
N6—C34	1.324 (4)	C27—C28	1.519 (5)
N6—C35	1.440 (5)	C29—C30	1.542 (5)
N6—H6	0.75 (4)	C29—H29A	0.9800
C1—C2	1.389 (5)	C29—H29B	0.9800
C1—H1	0.9400	C31—C33	1.381 (4)
C2—C3	1.360 (6)	C31—C32^ii^	1.385 (5)
C2—H2	0.9400	C31—H31	0.9400
C3—C4	1.410 (5)	C32—C33	1.387 (5)
C3—H3	0.9400	C32—H32	0.9400
C4—C12	1.397 (4)	C33—C34	1.500 (5)
C4—C5	1.432 (5)	C35—C36	1.533 (5)
C5—C6	1.350 (6)	C35—H35A	0.9800
C5—H5C	0.9400	C35—H35B	0.9800
C6—C7	1.420 (5)		
			
N1—Cu—N3	174.71 (11)	C15—C14—C13	118.7 (4)
N1—Cu—N4	93.74 (11)	C15—C14—H14	120.6
N3—Cu—N4	82.45 (11)	C13—C14—H14	120.6
N1—Cu—N2	81.30 (11)	C14—C15—C16	120.6 (3)
N3—Cu—N2	97.30 (11)	C14—C15—H15	119.7
N4—Cu—N2	118.90 (10)	C16—C15—H15	119.7
N1—Cu—O1	90.02 (11)	C24—C16—C15	117.6 (3)
N3—Cu—O1	94.44 (11)	C24—C16—C17	117.3 (4)
N4—Cu—O1	103.88 (12)	C15—C16—C17	125.1 (4)
N2—Cu—O1	136.69 (11)	C18—C17—C16	121.9 (4)
C30—O1—Cu	98.7 (3)	C18—C17—H17	119.0
C36—O5—H5	99 (4)	C16—C17—H17	119.0
H10A—O10—H10B	103 (7)	C17—C18—C19	122.1 (4)
H11A—O11—H11B	121.6	C17—C18—H18	119.0
H12A—O12—H12B	114 (8)	C19—C18—H18	119.0
H13A—O13—H13B	99 (7)	C23—C19—C20	118.0 (4)
C1—N1—C12	119.3 (3)	C23—C19—C18	117.5 (4)
C1—N1—Cu	126.8 (2)	C20—C19—C18	124.4 (4)
C12—N1—Cu	113.9 (2)	C21—C20—C19	119.9 (4)
C10—N2—C11	117.0 (3)	C21—C20—H20	120.1
C10—N2—Cu	132.6 (2)	C19—C20—H20	120.1
C11—N2—Cu	110.3 (2)	C20—C21—C22	118.4 (4)
C13—N3—C24	119.5 (3)	C20—C21—H21	120.8
C13—N3—Cu	127.8 (2)	C22—C21—H21	120.8
C24—N3—Cu	112.7 (2)	N4—C22—C21	123.5 (4)
C22—N4—C23	118.1 (3)	N4—C22—H22	118.2
C22—N4—Cu	132.7 (3)	C21—C22—H22	118.2
C23—N4—Cu	109.1 (2)	N4—C23—C19	122.0 (3)
C28—N5—C29	123.9 (3)	N4—C23—C24	117.4 (3)
C28—N5—H5N	121 (3)	C19—C23—C24	120.6 (3)
C29—N5—H5N	115 (3)	N3—C24—C16	121.2 (3)
C34—N6—C35	120.2 (3)	N3—C24—C23	118.3 (3)
C34—N6—H6	122 (3)	C16—C24—C23	120.6 (3)
C35—N6—H6	118 (3)	C27—C25—C26^i^	121.2 (3)
O7—N7—O9	120.3 (4)	C27—C25—H25	119.4
O7—N7—O8	119.2 (4)	C26^i^—C25—H25	119.4
O9—N7—O8	120.4 (4)	C27—C26—C25^i^	121.7 (3)
N1—C1—C2	121.7 (4)	C27—C26—H26	119.2
N1—C1—H1	119.2	C25^i^—C26—H26	119.2
C2—C1—H1	119.2	C25—C27—C26	117.1 (3)
C3—C2—C1	119.7 (3)	C25—C27—C28	124.2 (3)
C3—C2—H2	120.1	C26—C27—C28	118.6 (3)
C1—C2—H2	120.1	O3—C28—N5	121.5 (3)
C2—C3—C4	120.1 (3)	O3—C28—C27	121.8 (3)
C2—C3—H3	119.9	N5—C28—C27	116.8 (3)
C4—C3—H3	119.9	N5—C29—C30	113.6 (3)
C12—C4—C3	116.7 (3)	N5—C29—H29A	108.8
C12—C4—C5	118.8 (3)	C30—C29—H29A	108.8
C3—C4—C5	124.5 (3)	N5—C29—H29B	108.8
C6—C5—C4	121.6 (3)	C30—C29—H29B	108.8
C6—C5—H5C	119.2	H29A—C29—H29B	107.7
C4—C5—H5C	119.2	O2—C30—O1	123.1 (4)
C5—C6—C7	120.8 (3)	O2—C30—C29	120.4 (4)
C5—C6—H6C	119.6	O1—C30—C29	116.5 (4)
C7—C6—H6C	119.6	C33—C31—C32^ii^	120.4 (3)
C11—C7—C8	117.6 (3)	C33—C31—H31	119.8
C11—C7—C6	118.9 (3)	C32^ii^—C31—H31	119.8
C8—C7—C6	123.5 (3)	C31^ii^—C32—C33	120.5 (3)
C9—C8—C7	119.0 (3)	C31^ii^—C32—H32	119.7
C9—C8—H8	120.5	C33—C32—H32	119.7
C7—C8—H8	120.5	C31—C33—C32	119.0 (3)
C8—C9—C10	119.7 (3)	C31—C33—C34	116.3 (3)
C8—C9—H9	120.2	C32—C33—C34	124.6 (3)
C10—C9—H9	120.2	O6—C34—N6	120.8 (3)
N2—C10—C9	123.5 (3)	O6—C34—C33	121.7 (3)
N2—C10—H10	118.2	N6—C34—C33	117.4 (3)
C9—C10—H10	118.2	N6—C35—C36	112.1 (3)
N2—C11—C7	123.3 (3)	N6—C35—H35A	109.2
N2—C11—C12	116.4 (3)	C36—C35—H35A	109.2
C7—C11—C12	120.3 (3)	N6—C35—H35B	109.2
N1—C12—C4	122.5 (3)	C36—C35—H35B	109.2
N1—C12—C11	118.0 (3)	H35A—C35—H35B	107.9
C4—C12—C11	119.5 (3)	O4—C36—O5	124.3 (4)
N3—C13—C14	122.4 (3)	O4—C36—C35	125.6 (4)
N3—C13—H13	118.8	O5—C36—C35	110.0 (4)
C14—C13—H13	118.8		
			
C12—N1—C1—C2	−1.6 (5)	C23—N4—C22—C21	−0.5 (6)
Cu—N1—C1—C2	177.3 (3)	Cu—N4—C22—C21	−179.0 (3)
N1—C1—C2—C3	1.7 (6)	C20—C21—C22—N4	0.7 (6)
C1—C2—C3—C4	−0.2 (5)	C22—N4—C23—C19	0.6 (5)
C2—C3—C4—C12	−1.2 (5)	Cu—N4—C23—C19	179.4 (3)
C2—C3—C4—C5	178.3 (3)	C22—N4—C23—C24	178.9 (3)
C12—C4—C5—C6	1.1 (5)	Cu—N4—C23—C24	−2.2 (4)
C3—C4—C5—C6	−178.3 (3)	C20—C19—C23—N4	−0.9 (5)
C4—C5—C6—C7	−0.4 (5)	C18—C19—C23—N4	177.7 (3)
C5—C6—C7—C11	−1.3 (5)	C20—C19—C23—C24	−179.2 (3)
C5—C6—C7—C8	178.7 (3)	C18—C19—C23—C24	−0.6 (5)
C11—C7—C8—C9	−0.5 (5)	C13—N3—C24—C16	−0.2 (5)
C6—C7—C8—C9	179.5 (3)	Cu—N3—C24—C16	179.8 (3)
C7—C8—C9—C10	−0.5 (5)	C13—N3—C24—C23	−178.8 (3)
C11—N2—C10—C9	−0.2 (5)	Cu—N3—C24—C23	1.3 (4)
Cu—N2—C10—C9	176.2 (2)	C15—C16—C24—N3	1.0 (5)
C8—C9—C10—N2	1.0 (5)	C17—C16—C24—N3	−178.9 (3)
C10—N2—C11—C7	−0.9 (4)	C15—C16—C24—C23	179.5 (3)
Cu—N2—C11—C7	−178.1 (2)	C17—C16—C24—C23	−0.4 (5)
C10—N2—C11—C12	178.1 (3)	N4—C23—C24—N3	0.8 (5)
Cu—N2—C11—C12	0.8 (3)	C19—C23—C24—N3	179.1 (3)
C8—C7—C11—N2	1.3 (4)	N4—C23—C24—C16	−177.8 (3)
C6—C7—C11—N2	−178.7 (3)	C19—C23—C24—C16	0.6 (5)
C8—C7—C11—C12	−177.6 (3)	C26^i^—C25—C27—C26	0.0 (6)
C6—C7—C11—C12	2.4 (4)	C26^i^—C25—C27—C28	178.1 (3)
C1—N1—C12—C4	0.0 (4)	C25^i^—C26—C27—C25	0.0 (6)
Cu—N1—C12—C4	−178.9 (2)	C25^i^—C26—C27—C28	−178.3 (3)
C1—N1—C12—C11	−178.1 (3)	C29—N5—C28—O3	3.2 (6)
Cu—N1—C12—C11	2.9 (3)	C29—N5—C28—C27	−175.6 (3)
C3—C4—C12—N1	1.3 (4)	C25—C27—C28—O3	173.1 (4)
C5—C4—C12—N1	−178.2 (3)	C26—C27—C28—O3	−8.8 (5)
C3—C4—C12—C11	179.5 (3)	C25—C27—C28—N5	−8.2 (5)
C5—C4—C12—C11	0.0 (4)	C26—C27—C28—N5	170.0 (3)
N2—C11—C12—N1	−2.5 (4)	C28—N5—C29—C30	−104.5 (4)
C7—C11—C12—N1	176.5 (3)	Cu—O1—C30—O2	0.1 (4)
N2—C11—C12—C4	179.3 (3)	Cu—O1—C30—C29	−177.7 (3)
C7—C11—C12—C4	−1.7 (4)	N5—C29—C30—O2	25.4 (5)
C24—N3—C13—C14	−0.6 (6)	N5—C29—C30—O1	−156.7 (3)
Cu—N3—C13—C14	179.3 (3)	C32^ii^—C31—C33—C32	0.4 (5)
N3—C13—C14—C15	0.6 (6)	C32^ii^—C31—C33—C34	−179.2 (3)
C13—C14—C15—C16	0.1 (6)	C31^ii^—C32—C33—C31	−0.4 (5)
C14—C15—C16—C24	−0.9 (6)	C31^ii^—C32—C33—C34	179.1 (3)
C14—C15—C16—C17	179.0 (4)	C35—N6—C34—O6	0.6 (5)
C24—C16—C17—C18	0.3 (6)	C35—N6—C34—C33	−178.2 (3)
C15—C16—C17—C18	−179.6 (4)	C31—C33—C34—O6	−7.2 (4)
C16—C17—C18—C19	−0.3 (7)	C32—C33—C34—O6	173.3 (3)
C17—C18—C19—C23	0.5 (6)	C31—C33—C34—N6	171.6 (3)
C17—C18—C19—C20	179.0 (4)	C32—C33—C34—N6	−8.0 (5)
C23—C19—C20—C21	1.2 (6)	C34—N6—C35—C36	−81.3 (4)
C18—C19—C20—C21	−177.3 (4)	N6—C35—C36—O4	−5.3 (6)
C19—C20—C21—C22	−1.1 (6)	N6—C35—C36—O5	175.1 (3)

Symmetry codes: (i) −*x*+2, −*y*, −*z*+1; (ii) −*x*, −*y*+2, −*z*+2.

### Hydrogen-bond geometry (Å, º)

**Table:**

*D*—H···*A*	*D*—H	H···*A*	*D*···*A*	*D*—H···*A*
O5—H5···O11	0.87 (6)	1.84 (6)	2.665 (5)	159 (6)
O10—H10*A*···O2^iii^	0.83 (7)	2.06 (7)	2.878 (6)	168 (7)
O10—H10*B*···O7	0.85 (7)	2.24 (7)	3.033 (6)	155 (7)
O11—H11*A*···O8	0.85	2.00	2.848 (6)	179
O11—H11*B*···O1	1.00	1.68	2.643 (6)	160
O12—H12*A*···O7	0.79 (8)	2.32 (8)	3.024 (6)	149 (8)
O12—H12*B*···O13	0.80 (8)	2.07 (8)	2.856 (6)	170 (8)
O13—H13*A*···O12^iv^	0.75 (7)	2.05 (7)	2.757 (6)	157 (8)
O13—H13*B*···O6	0.81 (7)	2.05 (7)	2.851 (4)	170 (7)
N5—H5*N*···O8^v^	0.79 (4)	2.23 (5)	2.947 (4)	152 (4)
N6—H6···O3^vi^	0.75 (4)	2.26 (4)	2.995 (4)	167 (4)
C2—H2···O13	0.94	2.37	3.302 (5)	172
C3—H3···O10^iv^	0.94	2.44	3.358 (7)	165
C9—H9···O4^vii^	0.94	2.40	3.099 (5)	131
C13—H13···O2	0.94	2.42	3.031 (5)	122
C14—H14···O10^v^	0.94	2.60	3.414 (5)	145
C25—H25···O9^v^	0.94	2.53	3.354 (6)	147
C32—H32···O3^vi^	0.94	2.48	3.388 (4)	163
C35—H35*A*···O10^viii^	0.98	2.53	3.378 (5)	145

Symmetry codes: (iii) *x*, *y*+1, *z*; (iv) −*x*+1, −*y*+1, −*z*+2; (v) −*x*+1, −*y*+1, −*z*+1; (vi) *x*−1, *y*+1, *z*; (vii) *x*, *y*−1, *z*; (viii) *x*−1, *y*, *z*.
